# Constipation

**Published:** 1877-05-20

**Authors:** L. B. Anderson

**Affiliations:** Va.


### CONSTIPATION.

BY L. B. ANDERSON, of Va.

I have a venerable patient in his 87th year, who from early manhood has scarcely had an alvine evacuation oftener than once in fourteen days. He has enjoyed, nevertheless, remarkable exemption from disease. There is but little doubt, that his kidneys have performed for many years, more than their accustomed labor. And occasionally they have, within the last few years suffered from unusual irritation. His bladder also, has experienced the effect of the morbid secretion from the kidneys, to a considerable degree, within the last three years. During the past winter, and up to this time, he has suffered from a severe rheumatic affection of the hips, loins, bladder and spermatic cord. During this time he has taken large quantities of iodide and bromide of potassium, wine of colchicum, ipecac, cohosh, quinine, etc., and other agents occasionally having a decided cathartic tendency. But the habit of his life has not been broken, and only once in two weeks would he have an action of his bowels.

Some three weeks ago, I deemed it necessary, to relieve pain and cystic irritability at night, and thus procure rest, to give him at bed time, a pill composed of 1 grain of opium and 2 grains of gum camphor. In two days after he commenced their use, he had a free passage from his bowels, and had not, many days after, when I last saw him, failed to have a full evacuation every day, or at the most, every other day.

A very interesting and practical physiological and philosophical principle is involved in this fact, and I record it for the investigation and consideration of my medical brethren.

